# Interaction of *Tetracapsuloides bryosalmonae*, the causative agent of proliferative kidney disease, with host proteins in the kidney of *Salmo trutta*

**DOI:** 10.1007/s00436-015-4357-7

**Published:** 2015-02-07

**Authors:** Gokhlesh Kumar, Michael Gotesman, Mansour El-Matbouli

**Affiliations:** Clinical Division of Fish Medicine, Department for Farm Animals and Veterinary Public Health, University of Veterinary Medicine, Veterinärplatz 1, 1210 Vienna, Austria

**Keywords:** Salmonids, Myxozoa, Proliferative kidney disease, Protein interaction, Electrospray ionization mass spectrometry

## Abstract

*Tetracapsuloides bryosalmonae* (Myxozoa) is the causative agent of proliferative kidney disease in various species of salmonids which are found in Europe and North America. Less information about the interactions of *T. bryosalmonae* proteins with salmonid proteins during parasite development is known. In this study, anti-*T. bryosalmonae* monoclonal antibody-linked to *N*-hydroxysuccinimide-activated spin columns were used to purify parasite and host proteins from the kidneys of infected and non-infected brown trout (*Salmo trutta*) Linnaeus, 1758. The samples were next analyzed by electrospray ionization coupled to mass spectrometry to identify proteins that may be involved in the infection and proliferation of *T. bryosalmonae* within the brown trout host. A total of 6 parasite proteins and 40 different host proteins were identified in this analysis. The identified host proteins function in various processes, which include host defense, enzymatic, and structural components. In conjunction with modern molecular based tools, such siRNA, gene replacement, or gene disruption, this data can ultimately be used to develop novel control methods for *T. bryosalmonae*, based on the proteins or pathways identified in this study.

## Introduction

*Tetracapsuloides bryosalmonae* belongs to the phylum Myxozoa, class Malacosporea, and causes proliferative kidney disease (PKD) in various species of salmonids (Anderson et al. 1999). This parasite is found in Europe and North America and can lead to severe losses in rainbow trout (*Oncorhynchus mykiss*) Walbaum, 1792, and brown trout (*Salmo trutta*) Linnaeus, 1758; farms and the associated economic impact of this disease makes it an important factor for aquaculture (El-Matbouli and Hoffmann 2002). Additionally, PKD is suspected of contributing to the decline of wild brown trout and salmon populations, especially in Switzerland (Wahli et al. 2002). Spores develop in the kidney tubules of infected fish and are released via urine to infect freshwater bryozoans (Morris and Adams 2006, 2007; Grabner and El-Matbouli 2008). Proliferation of *T. bryosalmonae* induces granulomatous cellular response in the interstitial tissue which induces swelling of the kidney and spleen (Clifton-Hadley and Feist 1989).

Knowledge about protein interactions may be used to understand how parasites enter host cells and the process of parasite development during infection and help explain the selectivity of the parasite for the targeted tissue. However, very little is known about the interactions of *T. bryosalmonae* proteins with salmonid proteins during parasite entry or development. Protein purification using antibody-based methods can be used to study in vivo protein interactions (Tuxworth et al. 2005; Gotesman et al. 2011). Mass spectrometry has become a standard protocol in the identification of proteins and has become favored over the classical Edman degradation method (Cameron 2012). Electrospray ionization mass spectrometry (ESI-MS) is a highly sensitive form of MS used in the detection of samples that are at femtomolar concentrations in nanomole quantities (Ho et al. 2003). Recently, antibody-based protein purification followed ESI-MS was used to investigate Cyprinid herpesvirus 3 (CyHV-3) and host interactions in common carp (*Cyprinus carpio*) and identified several host defense proteins that may interact with CyHV-3 (Gotesman et al. 2013).

The objective of this study was to identify parasite and host protein interactions for *T. bryosalmonae* in the kidney of brown trout host by antibody-based protein purification followed by ESI-MS. The method of choice for this study was the use of antibody-based protein purification followed by ESI-MS to identify parasite–host protein interactions. From the identification of proteins in this study, there is an improvement in our understanding of parasite development in brown trout, and the identified proteins could potentially serve as targets for control of PKD in salmonids.

## Materials and methods

### Fish samples

Fish used for the present study originated from a previous experiment. Details of the experimental design and sampling procedure have been provided previously (Kumar et al. 2013, 2014, 2015). Briefly, brown trout were infected with free spores of *T. bryosalmonae* and maintained under laboratory conditions. Posterior kidneys were sampled from fish at different time points and tested for the presence of *T. bryosalmonae* by real-time PCR (qRT-PCR) and immunohistology using anti-*T. bryosalmonae* monoclonal antibody (MAB) P01 (Kumar et al. 2013).

### Tissue lysate preparation

Kidney samples (*n* = 8) collected at 8–10 weeks post exposure (wpe) were homogenized in a 1:1 ratio with a non-denaturing lysis buffer: 50 mM Tris–HCl (pH 8.0), 150 mM, NaCl, 20 mM ethylene diamine tetraacetic acid, 1 % Na-deoxycholate, 1 % Triton X-100 (Williams 2000), and protease inhibitor cocktail (100 μl inhibitor/ml of lysis buffer). Subsequently, lysate was vigorously vortexed and centrifuged at 14,000 *g* for 15 min. The supernatant was transferred to a sterile 1.5-mL tubes and re-centrifuged at 14,000 *g* for an additional 15 min. Supernatant from the second centrifugation for each fraction was separately used for affinity purification as described in the following sections.

### Western blotting

Western blot analysis was used to test specificity of anti-*T. bryosalmonae* MAB P01 (Aquatic Diagnostics Ltd) in both infected and non-infected kidney supernatant protein samples. Briefly, supernatant proteins were separated by electrophoresis on 12 % SDS-PAGE gel, and then proteins were transferred onto polyvinylidene difluoride membrane. The membrane was blocked, incubated with anti-*T. bryosalmonae* MAB, and later incubated with goat anti-mouse IgG HRP conjugate. After washing, the reactivity of antibody was confirmed by colorimetric detection using an Opti-4CN substrate (BIO-RAD).

### Preparation of monoclonal antibody-linked spin columns

Monoclonal antibody IgG1 isotype against *T. bryosalmonae* P01 was conjugated to *N*-hydroxysuccinimide-activated 33-mg-capacity agarose spin column according to the manufacturer’s instructions. *T. bryosalmonae* MABs recognize parasite surface antigens and bind to both extrasporogonic and sporogonic stages of parasite (Marin de Mateo et al. 1996). Anti-*T. bryosalmonae* MAB (200 μg) was resuspended into 400-μl phosphate-buffered saline (PBS) at pH 7.4 and incubated in agarose spin column overnight at 4 °C with mild shaking of 300 rpm in an Eppendorf Thermomixer Comfort. The spin columns were emptied and washed twice with PBS after overnight incubation. Subsequently, the spin columns were quenched with 1 M ethanolamine, pH 7.4, by incubation for 1 h at 12 °C with mild shaking. Finally, the spin columns were emptied by centrifugation and washed six times with PBS.

### Protein purification

Non-denatured tissue extracts from *T. bryosalmonae* infected and non-infected kidneys samples were separately incubated overnight at 12 °C with mild shaking at 300 rpm in monoclonal antibody-linked spin columns (previously described). Additionally, control spin columns (with or without antibody) were used to test for unspecific binding of proteins. The spin columns were washed three additional times after the flow of the washes was 0 at optical density at 280 (OD_280_), for a total of eight washes with PBS. The spin columns were subsequently eluted with 0.1 M glycine, pH 3.0, by incubation with mild shaking for 1 h at 12 °C, and the pH was neutralized by addition of 1 M Tris base, pH 8.0. The columns were next washed with PBS and eluted. Eluted fractions were concentrated using a dry vacuum concentrator.

### ESI-MS analysis

The samples, which consisted of the entire antibody-purified products originating from either infected or non-infected fish, were also separately analyzed by ESI-MS. In-solution digest using trypsin was performed for each sample. The resulting peptides were analyzed by LC-MS/MS using a nanoHPLC system coupled with the ion trap mass spectrometer. The instrument’s software was used to handle data acquisition and data processing. Peptides were searched with the mascot search engine (http://www.matrixscience.com) using proteins of fish, mammals, or myxozoan parasites in the NCBInr, UniProt, and Swiss-Prot and dbEST databases to identify proteins. The following parameters were used: enzyme trypsin, fixed modifications carbamidomethyl (C), variable modifications deamidation (NQ) and oxidation (M), peptide mass tolerance 5 ppm, fragment mass tolerance 0.4 Da, and with one missed cleavage site allowed. Proteins that have at least two unique peptides with a significant score (*P <* 0.01) and ion score cut-off of 20 in the mascot search were considered for protein identification. Electrospray ionization mass spectrometry analysis was performed by the DKFZ, The German Cancer Research Centre, Heidelberg, Germany.

## Results

### Western blotting

**Fig. 1:**
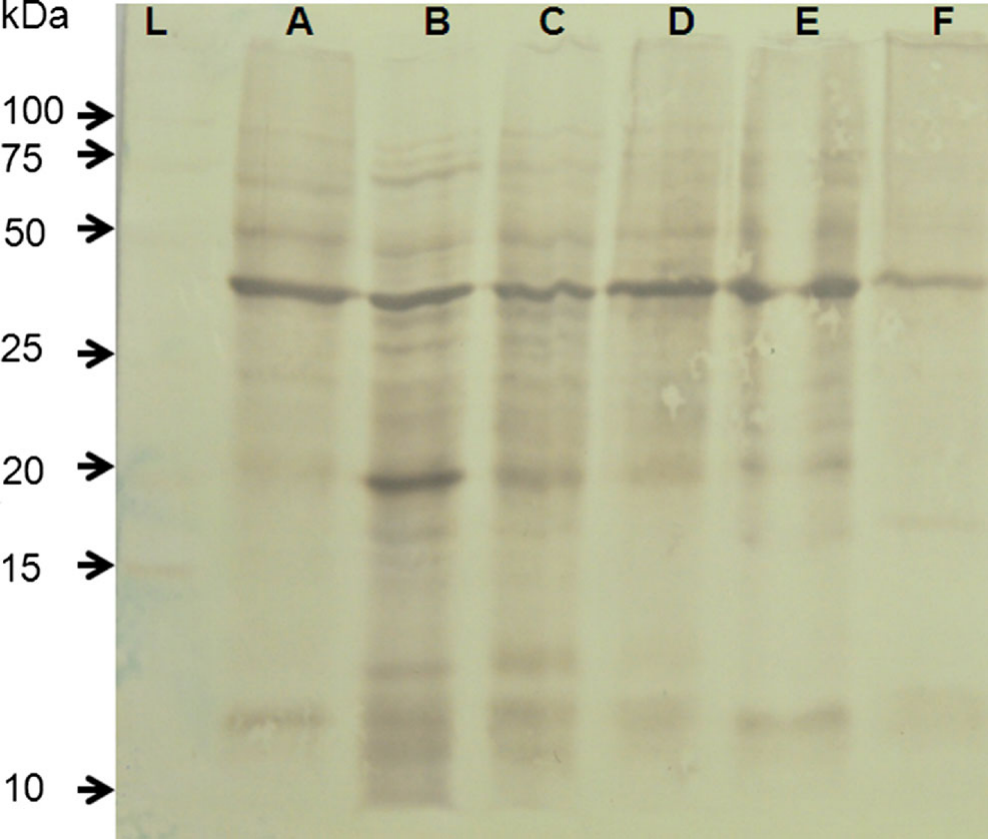
Western blot analysis of supernatant proteins from infected and non-infected kidneys of brown trout, *Salmo trutta. Lanes A*–*E*, supernatant proteins from infected kidney; *lane F*, supernatant proteins from non-infected kidney

In Western blotting, anti-*T. bryosalmonae* MAB detected differ blotting profiles in the kidney supernatants of infected brown trout. The differences were for major and minor protein bands in the kidney supernatants of infected brown trout and few protein bands in non-infected kidney (Fig. 1).

### ESI-MS analysis

A total of 6 parasite proteins and 40 different host proteins were identified in this analysis. The parasite proteins identified consisted of several structural proteins, such as actin and histones as well an enzymatic protein termed glyceraldehyde-3-phosphate dehydrogenase (G3DPH) and a small nuclear ribonuclear protein (Table 1). Of the 30 host proteins identified in the kidney of brown trout infected with *T. bryosalmonae*, 6 were also identified in the kidney of non-infected brown trout (Table 2) and the other 24 were unique to the infected brown trout (Table 3). Similarly, of 10 proteins identified in the kidney of non-infected brown trout, 4 were unique to non-infected brown trout (Table 3).

**Table 1:** Malacosporean parasite proteins identified in the kidneys of brown trout infected with *Tetracapsuloides bryosalmonae*

Protein description	Accession number	Mass (Da)	Score	Matches	Coverage (%)
Actin	gi|148491610	31449	90	6	18.7
Histone H3	gi|148491585	18822	84	6	9.4
Histone H2B	gi|148491596	18828	25	2	5.1
Hypothetical protein	gi|148491565	17407	28	2	5.7
Glyceraldehyde-3-phosphate dehydrogenase	gi|148492233	34089	24	2	2.2
Small nuclear ribonucleoprotein D1 polypeptide 16 kDa	gi|148491909	32913	29	2	1.7

**Table 2:** Overlapping host proteins identified in the kidneys of both infected and non-infected brown trout

Protein description	Accession number	Mass (Da)	Score	Matches	Coverage (%)
Hornerin precursor	gi|40795897	283111	239	9	7
Serpin B12	gi|17998551	46646	156	10	6
Annexin A2 isoform 2	gi|4757756	38808	214	9	6
Glyceraldehyde-3-phosphate dehydrogenase	gi|31645	36202	179	7	4
High choriolytic enzyme 1 precursor	gi|225705620	32596	85	3	2
Fatty acid-binding protein	gi|4557581	15497	187	10	4

**Table 3:** Unique proteins identified in the kidneys of brown trout

Protein description	Accession number	Mass (Da)	Score	Matches	Coverage (%)
24 proteins identified in the kidneys of brown trout infected with *Tetracapsuloides bryosalmonae*					
Epiplakin	gi|478538034	285127	77	3	2
Uncharacterized protein LOC101044508	gi|403308699	148282	262	14	7
Protein NLRC3-like	gi|348522660	82990	63	3	2
Band-6-protein	gi|535015	81637	227	10	6
Transglutaminase E3	gi|307504	77121	122	4	3
11S globulin-like protein	gi|18479082	59605	372	20	8
Xaa-Pro aminopeptidase	gi|488783751	53714	63	3	2
Uncharacterized protein LOC678611	gi|94536645	51128	327	31	7
Major facilitator transporter	gi|493287502	51089	62	2	2
Squamous cell carcinoma antigen	gi|239552	44564	93	5	3
Hypothetical protein BRAFLDRAFT	gi|260785919	42116	173	9	6
Hypothetical protein PANDA_012410	gi|281341802	29722	262	34	5
14-3-3 protein	gi|46326988	29216	62	2	2
Histone H2A-like	gi|528484029	27184	219	12	6
Trypsin 10 precursor	gi|84781771	26888	63	3	2
Protease, serine, 1 precursor	gi|16716569	26802	98	12	2
Unnamed protein product	gi|47225212	25650	223	10	6
Casein alphaS1	gi|225632	24477	139	6	3
Peroxiredoxin-1	gi|4505591	22324	86	4	2
Hemoglobin IV beta	gi|185135748	16243	111	4	3
Protein S100A9	gi|4506773	13291	86	5	3
Small proline-rich protein	gi|33842	10363	110	6	4
Cationic trypsinogen	gi|1616766	9200	80	3	2
Annexin A1	gi|4502101	3891	411	15	9
4 proteins identified in the kidneys of non-infected brown trout					
Myeloperoxidase precursor	gi|224613258	70130	97	4	3
Rhamnose-binding lectin STL1	gi|185134460	35198	120	3	1
Caspase-14 precursor	gi|6912286	27947	136	5	5
Dermcidin preproprotein	gi|16751921	11391	88	6	2

## Discussion

**Fig. 2:**
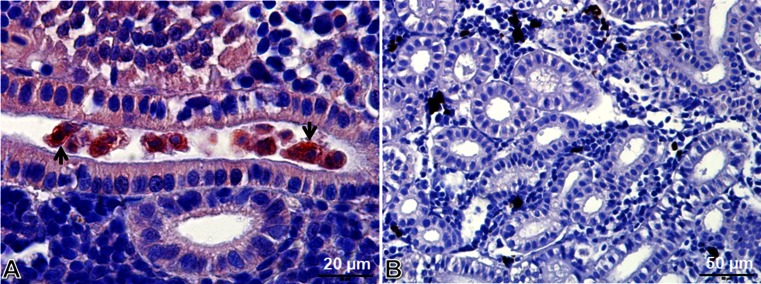
*Tetracapsuloides bryosalmonae* stages in kidney tissues of brown trout, *Salmo trutta*. **a** Intra-luminal sporogonic stages of parasite (*arrows*) and proliferation of the interstitial tissue can be seen in the kidney of brown trout. Tubule lumen filled with numerous intra-luminal sporogonic stages of the parasite. **b** Non-infected kidney of brown trout. Parasite stages were visualized by immunohistochemistry using anti-*T. bryosalmonae* monoclonal antibody and counterstained with hematoxylin

Parasite antigens are expressed and released into the host tissue during the sporogonic stages of the myxozoan parasite (Morris et al. 2004). Numerous intra-luminal sporogonic stages of *T. bryosalmonae* (Fig. 2a) were observed in the kidney of brown trout at 8–10 wpe along with low pre-sporogonic stages using immunohistological examination (Kumar et al. 2013). However, little is known about how the pathogen enters and proliferates inside the brown trout. Understanding host–pathogen interactions can be helpful to further elucidate the pathogenesis and proliferation of *T. bryosalmonae* within the salmonid host. For this study, antibody-based purification followed by ESI-MS was used to explore the protein interactions of *T. bryosalmonae* proteins with brown trout proteins during the developmental stages of the parasite. The antigen for the commercially available anti-*T. bryosalmonae* MAB P01 used in this study is unknown and also has not been fully characterized.

Of the 24 unique host proteins identified in the infected kidney sample, several have catalytic properties, such as transglutaminase E3, Xaa-Pro aminopeptidase, serine protease, and cationic trypsinogen. Some of the eclectic proteins include epiplakin which has been shown to have autoimmunogenic properties in humans (Fujiwara et al. 2001). Hemoglobin is the iron-containing oxygen-transport metalloprotein in erythrocytes (McMorrow et al. 1996). We found downregulation of hemoglobin in the kidney of infected brown trout (Kumar et al. 2014), which suggests that erythropoiesis was suppressed in the kidney. On the other hand, 26S protease regulatory subunit was upregulated in the kidney of infected brown trout (own unpublished data). Protein NOD-like receptor C3 is an intra-cellular protein that plays a role in the immune system and activation of T cells (Conti et al. 2005). The activation of T cells has been observed in the kidney of rainbow trout infected with *T. bryosalmonae* (Gorgoglione et al. 2013). Other important proteins, such as 14-3-3 and protein S100A9, were shown to be involved in either parasite or virus–host interactions in gilthead sea bream (*Sparus aurata*) and common carp, respectively (Davey et al. 2011; Ouyang et al. 2013). The 14-3-3 proteins are a family of conserved regulatory molecules that regulate a large spectrum of signaling pathways. The expression of 14-3-3 protein was downregulated in the head kidney of gilthead sea bream that was infected with myxozoan parasite, *Enteromyxum leei* (Davey et al. 2011). The aforementioned study suggests that the infection of *T. bryosalmonae* reduces the activities of signaling pathways that are essential for the induction of the brown trout immune system against parasite infection. S100A9, which is also known as migration inhibitory factor-related protein 14, is a calcium- and zinc-binding protein, and S100A9 plays a prominent role in the regulation of inflammatory processes and immune response of the host (Croce et al. 2009). The downregulation of S100A9 was observed in the head kidney of gilthead sea bream infected with *E. leei* by using microarray analysis (Davey et al. 2011). Therefore, the downregulation of S100A9 reduces inflammation in the kidney of infected brown trout and thereby supports developmental stages of *T. bryosalmonae*. These observations are in concordance with PKD signs in our previous study, in which we showed that the kidneys of rainbow trout, but not brown trout, were intensely swollen during parasite development (Kumar et al. 2013).

Six host proteins were identified in both infected and non-infected kidney samples (Table 2). Of the two enzymatic proteins identified, G3DPH and high choriolytic enzyme 1. G3DPH plays an important role in glycolysis and gluconeogenesis, and a major link between carbohydrate and lipid metabolisms (Ou et al. 2006). It is implicated in certain human neurological diseases such as Huntington’s, Alzheimer’s, and Parkinson’s diseases (Tatton et al. 2003). G3DPH is downregulated in the head kidney of gilthead sea bream infected with *E. leei*, suggesting that glycolysis and related metabolic pathways were affected in the kidney of fish during parasitic infection and may associate with the disease of myxozoan parasite. High choriolytic enzyme 1 precursor, activated high choriolytic enzyme 1 precursor, is a hatching enzyme that exhibits choriolysis and proteolysis activities in Japanese rice fish (*Oryzias latipes*) (Yasumasu et al. 1989). Additionally, of the six malacosporean parasite proteins identified in this study, the presence of histones and small nuclear ribonucleoprotein indicates that the parasite is continuously managing transcription during development (Baer and Rhodes 1983).

Because overlapping proteins were eluted in both the infected and non-infected samples, this suggests that the antibody may cross-react with some host proteins as seen in the Western blot analysis (Fig. 1). In previous immunohistological studies, this antibody binds specifically to unknown antigen expressed on the surface of *T. bryosalmonae* (Marin de Mateo et al. 1996), and also the exact nature of this cross-reaction in both Western blot and antibody-based purification is unknown. Additionally, identified proteins that were unique to non-infected tissue may be have been outcompeted from interacting with the antibody or may have been downregulated in infected tissue and therefore not detected in samples originating from infected tissue samples. A detailed study in the future would help to understand the cross-reaction of antibody and the nature of parasite antigen.

In conclusions, we identified host–parasite protein interactions for the proliferative kidney disease and serves as an explorative method that can be used in conjunction with qRT-PCR and microarray-based techniques to identify important proteins and pathways involved in the proliferation of the disease. This data can be used to develop novel control methods for *T. bryosalmonae*, based on the proteins or pathways identified in this study using RNA interference technology or other molecular based tools to differentially regulate genes implicated in PKD. Moreover, these proteins could be used for understanding the pathogenesis and defense mechanisms of other malacosporean species. Further research is needed to better understand how these proteins and their associated pathways are involved in the development of parasitic stages in the kidney of salmonids. Additionally, the identification in this study of so many different proteins clustered within a limited subset of functional pathways is in agreement with earlier reports of the promiscuous nature of protein interactions (Han et al. 2004; Agarwal et al. 2010). The results of this study open the door for identifying gene targets important for PKD and should be validated by other methods, such as yeast two-hybrid screening or fluorescently labeled protein microscopy and microarray analysis.

## References

[CR1] Agarwal S, Deane CM, Porter MA, Jones NS (2010). Revisiting date and party hubs: novel approaches to role assignment in protein interaction networks. PLoS Comput Biol.

[CR2] Anderson CL, Canning EU, Okamura B (1999). Molecular data implicate bryozoans as hosts for PKX (Phylum Myxozoa) and identify a clade of bryozoan parasites within the Myxozoa. Parasitology.

[CR3] Baer BW, Rhodes D (1983). Eukaryotic RNA polymerase II binds to nucleosome cores from transcribed genes. Nature.

[CR4] Cameron LC (2012). Mass Spectrometry Imaging: facts and perspectives from a non-mass spectrometrist point of view. Methods.

[CR5] Clifton-Hadley RS, Feist SW (1989). Proliferative kidney disease in brown trout *Salmo trutta:* further evidence of a myxosporean aetiology. Dis Aquat Org.

[CR6] Conti BJ, Davis BK, Zhang J, O'Connor W, Williams KL, Ting JP (2005). CATERPILLER 16.2 (CLR16.2), a novel NBD/LRR family member that negatively regulates T cell function. J Biol Chem.

[CR7] Croce K, Gao H, Wang Y, Mooroka T, Sakuma M, Shi C, Sukhova GK, Packard RR, Hogg N, Libby P, Simon DI (2009). MRP-8/14 is critical for the biological response to vascular injury. Circulation.

[CR8] Davey GC, Calduch-Giner JA, Houeix B, Talbot A, Ariadna Sitjà-Bobadilla A, Prunetd P, Pérez-Sánchezb J, Cairnsa MT (2011). Molecular profiling of the gilthead sea bream (*Sparus aurata* L.) response to chronic exposure to the myxosporean parasite *Enteromyxum leei*. Mol Immunol.

[CR9] El-Matbouli M, Hoffmann RW (2002). Influence of water quality on the outbreak of proliferative kidney disease-field studies and exposure experiments. J Fish Dis.

[CR10] Fujiwara S, Takeo N, Otani Y, Parry DA, Kunimatsu M, Lu R, Sasaki M, Matsuo N, Khaleduzzaman M, Yoshioka H (2001). Epiplakin, a novel member of the Plakin family originally identified as a 450-kDa human epidermal autoantigen. Structure and tissue localization. J Biol Chem.

[CR11] Gorgoglione B, Wang T, Secombes CJ, Holland JW (2013). Immune gene expression profiling of proliferative kidney disease in rainbow trout *Oncorhynchus mykiss* reveals a dominance of anti-inflammatory, antibody and T helper cell-like activities. Vet Res.

[CR12] Gotesman M, Hosein RE, Gavin RH (2011). MyTH4 and FERM have distinct roles that are essential for the function of a class XIV myosin in *Tetrahymena thermophila*. Cytoskeleton.

[CR13] Gotesman M, Soliman H, El-Matbouli M (2013). Antibody screening identifies 78 putative host proteins involved in Cyprinid herpesvirus 3 infection or propagation in common carp, *Cyprinus carpio* L. J Fish Dis.

[CR14] Grabner DS, El-Matbouli M (2008). Transmission of *Tetracapsuloides bryosalmonae* (Myxozoa: Malacosporea) to *Fredericella sultana* (Bryozoa: Phylactolaemata) by various fish species. Dis Aquat Org.

[CR15] Han JDJ, Bertin N, Hao T, Goldberg DS, Berriz GF, Zhang LV, Vidal M (2004). Evidence for dynamically organized modularity in the yeast protein–protein interaction network. Nature.

[CR16] Ho CS, Lam CW, Chan MH, Cheung RC, Law LK, Lit LC, Ng KF, Suen MW, Tai HL (2003). Electrospray ionisation mass spectrometry: principles and clinical applications. Clin Biochem Rev.

[CR17] Kumar G, Abd-Elfattah A, Saleh M, El-Matbouli M (2013). Fate of *Tetracapsuloides bryosalmonae* (Myxozoa) after infection of brown trout *Salmo trutta* and rainbow trout *Oncorhynchus mykiss*. Dis Aquat Org.

[CR18] Kumar G, Abd-Elfattah A, El-Matbouli M (2014). Differential modulation of host genes in the kidney of brown trout *Salmo trutta* during sporogenesis of *Tetracapsuloides bryosalmonae* (Myxozoa). Vet Res.

[CR19] Kumar G, Abd-Elfattah A, El-Matbouli M (2015). Identification of differentially expressed genes of brown trout (*Salmo trutta*) and rainbow trout (*Oncorhynchus mykiss*) in response to *Tetracapsuloides bryosalmonae* (Myxozoa). Parasitol Res.

[CR20] Marin de Mateo M, George J, Morris D, Kent ML (1996). Comparative studies of PKX and *Sphaerospora* spp. from salmonids using lectin and monoclonal antibody staining techniques. J Fish Dis.

[CR21] McMorrow T, Wagner A, Deryckere F, Gannon F (1996). Structural organization and sequence analysis of the globin locus in Atlantic salmon. DNA Cell Biol.

[CR22] Morris DJ, Adams A (2006). Transmission of *Tetracapsuloides bryosalmonae* (Myxozoa: Malacosporea), the causative organism of salmonid proliferative kidney disease, to the freshwater bryozoan *Fredericella sultana*. Parasitology.

[CR23] Morris DJ, Adams A (2007). Sacculogenesis and sporogony of *Tetracapsuloides bryosalmonae* (Myxozoa: Malacosporea) within the bryozoan host *Fredericella sultana* (Bryozoa: Phylactolaemata). Parasitol Res.

[CR24] Morris DJ, El-Matbouli M, Adams A (2004). Extensive release of an antigen associated with the sporogonic stages of *Myxobolus cerebralis* (Myxozoa: Myxosporea) is detected by a heterologous antibody raised to *Tetracapsuloides bryosalmonae* (Myxozoa: Malacosporea). Folia Parasitol.

[CR25] Ou X, Ji C, Han X, Zhao X, Li X, Mao Y, Wong LL, Bartlam M, Rao Z (2006). Crystal structures of human glycerol 3-phosphate dehydrogenase 1 (GPD1). J Mol Biol.

[CR26] Ouyang P, Rakus K, Boutier M, Reschner A, Leroy B, Ronsmans M, Fournier G, Scohy S, Costes B, Wattiez R, Vanderplasschen A (2013). The IL-10 homologue encoded by cyprinid herpesvirus 3 is essential neither for viral replication in vitro nor for virulence in vivo. Vet Res.

[CR27] Tatton W, Chalmers-Redman R, Tatton N (2003). Neuroprotection by deprenyl and other propargylamines: glyceraldehyde-3-phosphate dehydrogenase rather than monoamine oxidase B. J Neural Transm.

[CR28] Tuxworth RI, Stephens S, Ryan ZC, Titus MA (2005). Identification of a myosin VII-talin complex. J Biol Chem.

[CR29] Wahli T, Knuessel R, Bernet D, Segner H, Pugovkin D, Burkhardt-Holm P, Escher M, Schmidt-Posthaus H (2002). Proliferative kidney disease in Switzerland: current state of knowledge. J Fish Dis.

[CR30] Williams NE, Asai DJ, Forney JD (2000). Immunoprecipitation procedures. Methods in cell biology: *Tetrahymena thermophila*.

[CR31] Yasumasu S, Luchi I, Yamagami K (1989). Purification and partial characterization of high choriolytic enzyme (HCE), a component of the hatching enzyme of the Teleost, *Oryzias latipes*. J Biochem.

